# To Bind or to Let Loose: Effectiveness of Sodium Polystyrene Sulfonate in Decreasing Serum Potassium

**DOI:** 10.1155/2012/940320

**Published:** 2012-12-27

**Authors:** Shaifali Sandal, Hatim Karachiwala, John Noviasky, Dongliang Wang, William C. Elliott, David F. Lehmann

**Affiliations:** ^1^Division of Nephrology, University of Rochester Medical Center, 601 Elmwood Avenue, P.O. Box 675, Rochester, NY 14642, USA; ^2^Division of Oncology, Dalhousie University, Room 457A, Bethune Building, 1276 South Park Street Halifax, Nova Scotia, Canada B3H 2Y9; ^3^Department of Pharmacy, SUNY Upstate Medical University, 750 East Adams Street, Syracuse, NY 13210, USA; ^4^Departments of Public Health and Preventive Medicine, SUNY Upstate Medical University, 750 East Adams Street, Syracuse, NY 13210, USA; ^5^Department of Medicine, SUNY Upstate Medical University, 750 East Adams Street, Syracuse, NY 13210, USA

## Abstract

*Background*. The use of sodium polystyrene sulfonate in decreasing serum potassium has recently been questioned due to the lack of documented effectiveness. *Methods*. A retrospective cohort analysis of all hospitalized patients who received sodium polystyrene sulfonate over four months was performed. The change in serum potassium was noted over a period of 24 hours. Patients who received any other form of potassium-altering drug or treatment were excluded. *Results*. The administration of sodium polystyrene sulfonate reduced serum potassium by 16.7% (*P* < 0.001) as compared to the baseline serum potassium over a period of 24 hours. During this same time, no change in serum creatinine was identified (*P* = 0.73). In addition, there was no correlation between potassium and creatinine change (*r* ^2^ = 0.0004 and *P* = 0.99). Patients with higher initial serum potassium (≥5.6 mEq/L) reduced their potassium concentration 4% more than those with initial serum potassium of <5.6 mEq/L; however, this reduction did not reach statistical significance (*P* = 0.32). There was no significant difference in the effectiveness of 15 gm and 30 gm resin preparation (*P* = 0.54). Thirteen deaths were noted in our cohort, of which one death was due to ischemic colitis. *Conclusion*. We conclude that sodium polystyrene sulfonate is effective in lowering serum potassium.

## 1. Introduction

Sodium polystyrene sulfonate (SPS) is a cation exchange resin used for the treatment of hyperkalemia. SPS use has been associated with rare but serious gastrointestinal complications such as rectal stenosis, colonic necrosis, ulceration, and perforation [1–19]. Despite its widespread use as a treatment modality for hyperkalemia, there is a paucity of data on SPS effectiveness in lowering serum potassium in human subjects. Due to these considerations, a recent review stated SPS use to be “largely unproven and potentially harmful” [20].

In light of these issues, we conducted a retrospective cohort study of patients at the Upstate Medical University who received SPS in this 419 bed, tertiary care medical center with a 4 bed unit for inpatient hemodialysis. The study was aimed to identify whether serum potassium decreased after administration of SPS over a period of 24 hours relative to the initial serum potassium. In addition, the effectiveness of 15 gm and 30 gm of SPS preparations was compared. A literature review was also conducted to identify patients who may be at risk for complications after SPS use.

## 2. Methods

A retrospective cohort study of hospitalized patients aged over 18 years was conducted who received SPS during their hospitalization. All patients received one or more doses of oral SPS in the first quarter of 2010 (between January 1, 2010 and April 30, 2010). Timing and dose of SPS given were captured by computerized billing data generated at time of dispensing. In those patients that received more than one dose of SPS within 24 hours, potassium measurements after the second SPS dose were excluded. The SPS suspension used was a commercially available, premixed cherry-flavored suspension containing 15 gm of SPS, 21.5 mL of sorbitol solution (equivalent to approximately 20 gm of sorbitol) and 0.18 mL (0.3%) of alcohol per 60 mL of suspension. Adverse events of SPS administration were identified by review of all discharge summaries written after administration of SPS.

Patients were excluded if they received hemodialysis, peritoneal dialysis, or continuous venovenous hemofiltration. In addition, patients were excluded if the following drugs were administered or dose changed over a period of 24 hours: diuretics, beta-adrenergic agonists/antagonists, potassium supplements, modulators of the renin-angiotensin-aldosterone system, insulin products. Patients were excluded if potassium specimen was hemolyzed. Hyperkalemia was defined as initial potassium levels greater than 5 mEq/L and initial levels greater than 7 mEq/L were excluded for data analysis as they were often given multiple modalities of potassium excretion. Adverse event review was performed in patients excluded from data analysis.

The primary outcome was the proportional change of potassium level. Each potassium level was utilized to check change in potassium over time. Also checked was maximal change in potassium by time intervals and, change in potassium relative to initial potassium and dose of SPS administered in grams (i.e., 15 gm or 30 gm). In order to determine influence of renal function, changes in creatinine level was correlated with changes in potassium levels.

The initial potassium served as the reference value for each patient and was compared with up to four follow-up measurements. The proportional potassium change was measured as the difference between the initial potassium and the follow-up potassium divided by the initial potassium. To minimize effect of regression to the mean, the proportional change was examined as those with higher baseline potassium level tend to decrease the potassium level more. The proportional change in potassium was fitted by a linear mixed model with time, the dose of SPS and the initial potassium as the predictor variables after a manual model selection process. Linear mixed model has been widely used to analyze longitudinal data. It provides reliable handling of the between- and within-subject variability, thus gaining more statistical power [21].

Follow-up potassium measurements were divided into five intervals, that is, 0–4 hr, 4.5–8 hr, 8.5–12 hr, 12.5–18 hr and >18 hr. A random intercept term was added to the model in order to account for the correlation structure between repeated measures within subjects. A similar procedure was also applied to examine the change in creatinine concentration. Other than demonstrate the independence of potassium and creatinine changes using the above mentioned model, a Pearson correlation coefficient was also calculated after collapsing observations from each patient and *P* value was calculated by Fisher's z transformation. All statistical tests were based at the significance level of 0.05. Data analyses were performed using SAS, version 9.2, statistical software (SAS Institute, Cary, NC).

## 3. Results

**Figure 1:**
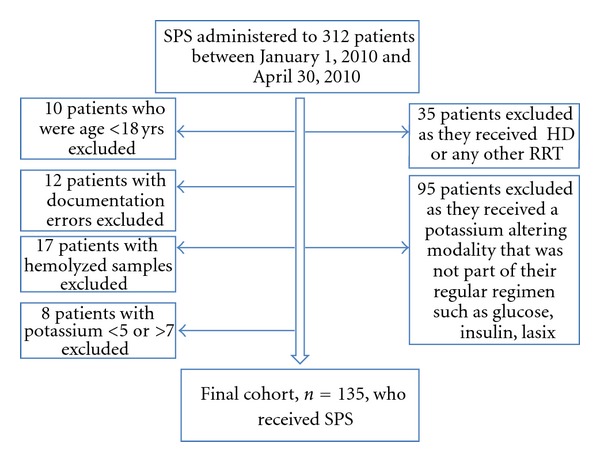
Study cohort.

**Figure 2:**
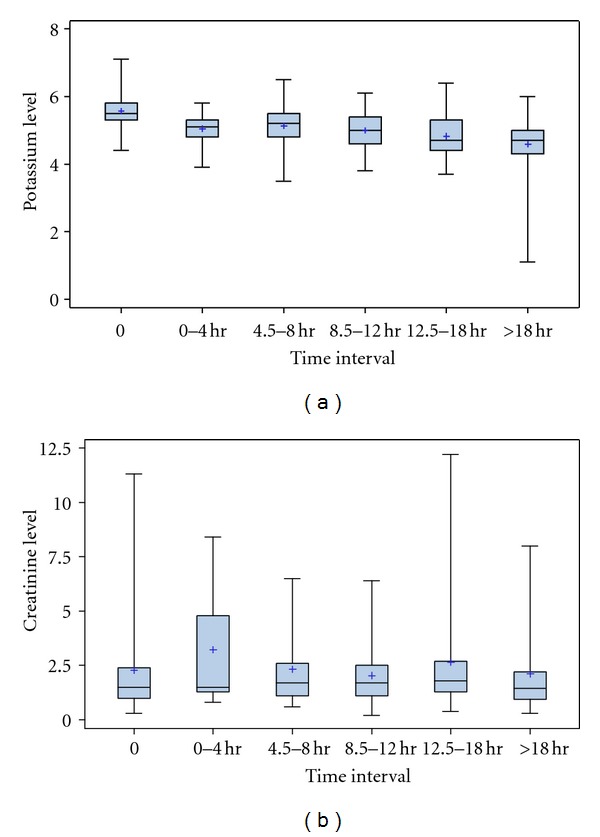
(a) The serum potassium values in mmol/L at the various time intervals. (b) The serum creatinine values in mg/dL at the various time intervals.

During the time period reviewed, SPS was administered 312 times to 171 patients. For the purpose of this study, each dose administered was treated as a separate SPS administration. 37% patients were women and 63% were men. 135 occurrences were obtained as summarized in Figure 1. The initial mean potassium was 5.59 ± 0.45 mmol/L and creatinine 2.34 ± 1.95 mg/dL.  Figure 2 demonstrates the distribution of the absolute potassium and creatinine values relative to time.

**Figure 3:**
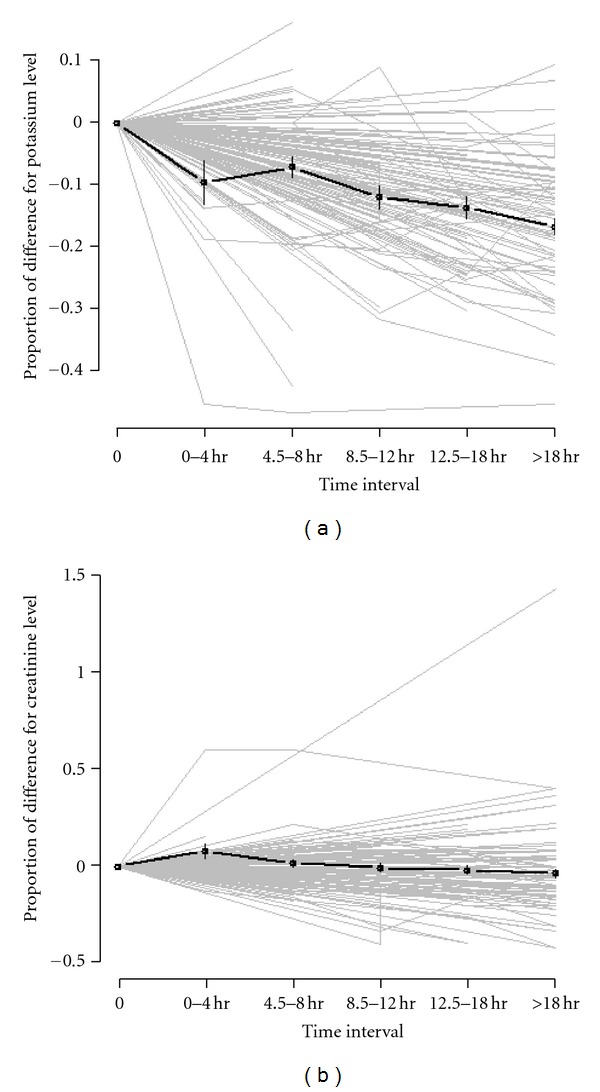
(a) Potassium versus time: proportion of change in serum potassium concentration [(*K*_*t*_ − *K*_0_)/*K*_0_] was fitted by a linear mixed model with the time of repeat potassium measurement as the predictor variable. A statistically significant decrease was noted in the proportion of change of potassium for time intervals 0–4 hr, 4.5–8 hr, and 8.5–12 hr (*P* value = 0.007, <0.0001, <0.0001, resp.). (b) Creatinine versus time: proportion of change in serum creatinine concentration [(*C*_*t*_ − *C*_0_)/*C*_0_] was fitted by a linear mixed model with the time of repeat creatinine measurement as the predictor variable. No statistically significant difference was noted in the proportion of change in creatinine in any of the time intervals (the *K* at *t*_0_ will be referred to as *K*_0_ and the follow-up *K* at time *t* will be referred to as *K*_*t*_. The *C* at *t*_0_ will be referred to as *C*_0_ and the follow up *C* at time *t* will be referred to as C_*t*_).

In these 135 occurrences, after administration of SPS, overall potassium decreased 16.7% (*P* < 0.001) as compared to the baseline measurement (Figure 3(a)). Potassium continued to decline over 24 hours and reached its minimum during 18 to 24 hours interval; however, there was no significant change in potassium between 12.5–18 hours and >18 hours intervals (*P* = 0.147). No change was noted in the proportion of change in creatinine concentration during the same time period (*P* = 0.73) (Figure 3(b)). There was no correlation between the change in serum potassium and change in serum creatinine (*r* ^2^ = 0.0004 and *P* = 0.99).

The initial potassium was divided into two categories (<5.6 mmol/L or ≥5.6 mmol/L). Those patients with higher initial potassium (≥5.6 mmol/L) decreased their potassium concentrations 4% more than those with initial potassium of <5.6 mmol/L, but this was not statistically significant (*P* = 0.32). There was no statistically significant difference in the effectiveness of 15 gm versus 30 gm of SPS preparation (*P* = 0.54).

Thirteen deaths were reported in our cohort. Of the 13 deaths, only one discharge summary identified ischemic colitis as one of the many causes of death but did not mention SPS as the etiology. This patient was a 59-year-old woman who had a past medical history of end stage renal disease on peritoneal dialysis and presented with non-ST elevation myocardial ischemia, lower extremity cellulitis, and peritonitis. She was hypotensive and in the intensive care unit. She was noted to be hyperkalemic and given SPS. She complained of abdominal pain within 24 hours of SPS administration and CT scan was demonstrative of ischemic colitis. She clinically deteriorated and eventually was made comfort care and died 7 days after administration of SPS. Unfortunately, post-mortem examination was not performed.

## 4. Discussion

Medical use of synthetic cation-exchange resins was reported in 1946 [20]. Despite the widespread use of SPS since, limited data exists on their effectiveness. Indeed, utility rests on studies done on animals and case series, case reports, and abstracts on human subjects [22–27]. Based on this data, a review article disputed the utility of SPS in lowering serum potassium [20]. For all of these considerations a formal study was conducted to study the effectiveness of SPS in lowering serum potassium. 

Our study demonstrates that SPS administration is followed by a statistically significant decrease in potassium levels in patients who did not receive any other form of potassium decreasing modality or any form of renal replacement therapy. No significant correlation was noted between change in serum potassium and serum creatinine. Thus, the decrease in potassium was not due to recovery of renal function and that SPS administration leads to a statistically significant decrease in the serum potassium level. These findings are similar to another study that showed normalization of serum potassium levels in 94% of the study patients [28]. Our study showed an overall potassium decrease of 16.7% (*P* < 0.001) as compared to the baseline measurement. The study by Kessler et al., showed a 15.1% and 17.2% decrease in the mean potassium concentration in patients receiving 15 gm and 30 gm of SPS, respectively [28]. 

Theoretical cation binding capacity of SPS or the Emax is 3.1 meq of potassium per gram of SPS in vitro and 0.8 mEq per gram of SPS in vivo [22, 24]. Our study did not show that more grams of SPS would cause higher reductions in potassium level. Based on our findings, a plateau appears to occur at 15 gm. This is an important implication because SPS may cause dose related intolerance. This is in contrast to the findings previously reported perhaps because none of our patients were given 45 gm or 60 gm of SPS [28]. Serum potassium levels decreased more if the initial potassium is higher; however, this was not statistically significant. 

Case reports of life-threatening adverse effects of SPS are most often found in pediatric patients, postsurgical patients, and transplant patients. The incidences of the potentially life threatening complications are estimated to be 0.14%–0.27% in those prescribed SPS compared to 0.07% in those not prescribed SPS [7, 29]. Postoperative patients are at a comparatively higher risk (1.8%) likely due to decreased bowel motility due to constipation, medications, ileus and mechanical obstruction which could increase the contact time of SPS with the bowel mucosa and predispose to damage [7, 14, 30]. Changes in the mucosal blood flow, hypotension, and hypovolemia could also predispose to increased colon injury [6, 10, 15]. Renal transplant patients are also considered high risk [31]. Patients receiving SPS may be at risk for intestinal necrosis from other causes, and studies showing direct causality are lacking [11, 32]. Some other lesser known risk factors that could predispose to SPS mediated intestinal injury are previous abdominal surgery, elevated renin, immunosuppression, antibiotic therapy, preceding irradiation, diverticular disease, coagulation disorder, opportunistic infections, and uremia [10, 12, 13, 31, 32]. Although a case series examining the surgical pathology samples of patients given SPS did identify that even non-postoperative patients and patients without significant vascular compromise are potentially at risk for gastrointestinal injury [33].

A literature review of all the adult case reports in the English language of SPS related complications was conducted (see Table I (S1) in Supplementary Material available online at doi:10.1155/2012/940320 summarizing these cases [1–19]). Nearly all patients who suffered catastrophic complications, including the patient in our cohort who had ischemic colitis, had the risk factors mentioned above. 

Hyperkalemia is a dangerous medical condition requiring prompt management and all available modalities have their own set of limitations and adverse effects [32]. We conclude that SPS is an effective modality to reduce serum potassium. We propose judicious SPS use in patients who have any of the major risk factors identified above and to have a high index of suspicion for gastrointestinal complications if clinical deterioration occurs.

## 5. Limitations

The potential limitations of our study are as follows. Given the retrospective cohort design the presence of other confounders cannot be excluded such as dietary changes. Also, the utility of the results in other cohorts might not be robust such as smaller centers and patients with severe hyperkalemia as these patients are often managed with multiple potassium decreasing modalities and hence were excluded from the study. Additionally, a lack of a control study also limits the validity of our results. One of the major concerns with SPS is whether it is the laxative or sorbitol effect that decreases potassium. This could not be determined with our study design as our SPS preparation contains sorbitol. We did not explore other adverse effects that patients may experience with SPS such as nausea and abdominal discomfort. We also did not explore the cause of hyperkalemia in our patient population. 

## Supplementary Material

Supplementary Table: I summarizes all adult case reports in the English language of Sodium Polystyrene Sodium related complications except the paper by Rashid et al as the authors have already done that in Table 1 of their paper. Supplementary table 1 summarizes the patient's history provided, complications and the intervention/s done. Nearly all patients who suffered catastrophic complications had the risk factors mentioned in the manuscript.
