# Supplementary material for: To Bind or to Let Loose: Effectiveness of Sodium Polystyrene Sulfonate in Decreasing Serum Potassium
Source: Int J Nephrol. 2012 Dec 27;2012:940320. doi: 10.1155/2012/940320 (PMC3576716; doi:10.1155/2012/940320)
Supplement: Supplementary file 1 — Supplementary Table: I summarizes all adult case reports in the English language of Sodium Polystyrene Sodium related complications except the paper by Rashid et al as the authors have already done that in Table 1 of their paper. Supplementary table 1 summarizes the patient's history provided, complications and the intervention/s done. Nearly all patients who suffered catastrophic complications had the risk factors mentioned in the manuscript. [file 940320.f1.pdf]

Supplemental Table I: Summary of case reports of SPS associated intestinal injury [1-19]

Paper	Age/sex	Patient description	Complication	Intervention/outcome
Lillemoe (1987)	15?	Transplant nephrectomy, AV shunt repair	Colonic necrosis, sepsis, necrotising fascitis	Colectomy/death
	40f	Nephrectomy, appendectomy	Transmural infacrction of colon	Right hemicolectomy
	52m	Coronary artery bypass graft	Mucosal and transmural infarction of the colon and cecum	Total abdominal colectomy/death
	34m	Transplant nephrectomy	Necrosis of the right and transverse colon and terminal ileum	Hemicolectomy, ileal resection/death
	31m	aortic valve replacement, multiorgan failure	Colonic necrosis and perforation	?/Death due to cardiac arrest
Wooton (1989)	48m	Renal transplant	Necrotic transverse colon	Colectomy
Gerstmann (1992)	43m	Transplant nephrectomy	Necrotic cecum and 2 frank perforations	Right colectomy
	42m	Cardiac transplantation/renal failure	Inflamed, edematous small bowel and right colon	Cecostomy tube placement
Scott (1993)	48m	Renal transplant	Within hours colonic necrosis	Colectomy
Roy-chaudhary (1997)	42f	Surgical revision of left acetabular graft	Gastric and ileocecal toxicity/serpiginous ulcers	Surgery
Gardiner (1997)	66m	Aortic valve replacment,cardiac arrest, multiorgan failure	Acute transmural inflammation	?/death
	71f	Chronic lower GI bleed, uremia	Acute transmural inflammation and focal hemhorrhage	Right hemicolectomy/death
Dardik (2000)	61m	Clostridium difficile colitis	Ischemic colitis	Transverse colectomy
Rogers (2001)	55m	Compartment syndrome with fasciotomies, multiorgan failure	Colonic necrosis	Colectomy
Cheng (2002)	53f	25% total body surface area burns, grafting surgery	Colonic necrosis/perforation, sepsis, multiorgan failure	Colectomy/Death
Kelsey (2003)	79m	Occlussion of vascular access with arteriogram, opiod use	Ischemic necrosis, perforation of the colon	Right ileocolectomy
Chatelain (2007)	46m	Motor vehical accident requiring thoracotomy and laporotomy	Ischemic colitis, sigmoid and rectal stricture	Hartmann's procedure,resection of the stricture
Shioya (2007)	77f	Diabetic ketoacidosis, duodenal ulcer hemhorrhage	Colonic ulcer and stenosis, 1 year later sigmoidovesical fistula	1 year later sigmoidectomy
Pusztaszeri (2007)	87m	Occlusion of vascular access	Jejunal diverticulitis	Resection of jejunum
Bomback (2009)	56f	Gastritis, given opiod, colon polyp	Colonic injury	Bi-directional endoscopy
Thomas (2009)	64f	Multiorgan failure, lower limb ischemia	Colonic necrosis	Colonoscopy
Joo (2009)	34m	Intracerebral hemorrhage with Burr hole	Acute colitis, hematochezia	2 sigmoidoscopies/death
Trottier (2009)	24f	Barbituric coma, ileus	Transmural colon necrosis, abdominal compartment syndrome	Right hemicolectomy, resection of ileum
Chou (2011)	30m	Lower gastrointestinal bleed	Colonic necrosis	Colonoscopy

This table summarizes all the case reports, except the paper by Rashid et al. as he has already summarized the clinical cases in table 1 of his paper [19].
